# Charge and discharge profiles of repurposed LiFePO_4_ batteries based on the UL 1974 standard

**DOI:** 10.1038/s41597-021-00954-3

**Published:** 2021-07-02

**Authors:** Hsien-Ching Chung

**Affiliations:** 1 Masterhold International Co., Ltd., New Taipei City, 231036 Taiwan; 2 Super Double Power Technology Co., Ltd., Changhua City, Changhua County 500042 Taiwan

**Keywords:** Electrical and electronic engineering, Renewable energy

## Abstract

Owing to the popularization of electric vehicles worldwide and the development of renewable energy supply, Li-ion batteries are widely used from small-scale personal mobile products to large-scale energy storage systems. Recently, the number of retired power batteries has largely increased, causing environmental protection threats and waste of resources. Since most of the retired power batteries still possess about 80% of their initial capacity, their second use becomes a possible route to solve the emergent problem. Safety and performance are important when using these second-use repurposed batteries. Underwriters Laboratories (UL), a global safety certification company, published the standard for evaluating the safety and performance of repurposed batteries, i.e., UL 1974. In this work, the test procedures are designed according to UL 1974, and the charge and discharge profile datasets of the LiFePO_4_ repurposed batteries are provided. Researchers and engineers can use the characteristic curves to evaluate the quality of the repurposed batteries. Furthermore, the profile datasets can be applied in the model-based engineering of repurposed batteries, e.g., fitting the variables of an empirical model or validating the results of a theoretical model.

## Abstract (summary)

**Table:** 

Measurement(s)	Charge and discharge profiles
Technology Type(s)	Two-tier DC load method

Machine-accessible metadata file describing the reported data: 10.6084/m9.figshare.14495604

## Background & Summary

The electrical energy storage system (EESS) is the capture of electrical energy produced at one time for use at a later time. The storage process involves converting electrical energy from forms that are difficult to store to forms that are more conveniently or economically storable, such as chemical, gravitational potential, elevated temperature, latent heat, and kinetic forms. The history of EESSs can be traced back to the early days of power generation, at the turn of the 20th century, where power stations were often shut down overnight, with lead-acid batteries supplying the residual loads on the direct current networks^1^. To overcome the temporary power shortage, many electrical energy storage technologies have been developed, such as pumped hydroelectric storage^2,3^, battery^4–7^, capacitor and supercapacitor^8–10^, compressed air energy storage^11–13^, flow battery^14–16^, fuel cell^17–19^, solar fuel^20–23^, superconducting magnetic energy storage^24–27^, flywheel^28^, and thermal energy storage^29^. Up to now, the pumped hydroelectric storage remains the main way for utility-scale electricity storage. This well-established technology has been commercially deployed since the 1890s^3^.

The development of renewable energy supply (mainly wind and solar photovoltaic) and electric vehicle (EV) industries advance the application of Li-ion batteries from small-scale 3 C (computing, communication, and consumer) products to large-scale battery energy storage systems (BESSs) and high-power mobile energy sources. The Li-ion battery exhibits the advantage of electrochemical energy storage, such as high power density, high energy density, very short response time, and suitable for various size scales (from 3 C to utility usages). For example, the installation of the world’s largest Li-ion battery (100 MW, 129 MWh from Tesla and Neoen) has been completed in South Australia in 2017^30,31^. After several weeks, when the coal-fired Loy Yang power plant in Victoria failed, leading to a power shortage, the backup battery kicked in and delivered as much as 100 MW into the national electricity grid in just 140 ms^32,33^, responding even more quickly than the coal-fired backups that were supposed to provide emergency power. That shock absorber-type and emitter-type capacities help us to stop a blackout that would otherwise occur. The batteries are capable of providing inertia services and rapid frequency responses (e.g., frequency control ancillary services; FCAS) to the grid^34–36^. Large-scale batteries begin to show their roles in supply electric networks since then.

Accompanied by the vigorous promotion of commercialization and the popularization of electric vehicles worldwide^37^, the Li-ion batteries are largely used, causing fundamental research, industrial development, as well as standard and policymaking in the field of Li-ion power batteries. Recently, the elimination of power batteries has largely increased, causing environmental protection threats and waste of resources. About 100–120 GWh of EV batteries will be retired by 2030^37^. Therefore, recycling and reutilization of such retired batteries have been promoted^38,39^. Some retired power batteries remain possessing about 80% of their initial capacity^40–43^. So they can be repurposed and utilized once again, for example, to serve the batteries in the stationary energy storage system^44–47^. Governments in various countries have acknowledged this emergent issue and prepared to launch their policies to deal with the recovery and reuse of repurposed batteries, such as coding principles, traceability management system, manufacturing factory guidelines, dismantling process guidelines, residual energy measurement, federal and state tax credits, rebates, and other financial support^48–50^.

Safety and performance are important in using the repurposed batteries. Underwriters Laboratories (UL), a global safety certification company established in 1894, published the standard for evaluating the safety and performance of repurposed batteries in 2018, i.e., UL 1974^51,52^. In this work, the charge and discharge profiles of lithium iron phosphate repurposed batteries are measured based on UL 1974. The lithium iron phosphate battery (LiFePO_4_ battery) or lithium ferrophosphate battery (LFP battery), is a type of Li-ion battery using LiFePO_4_ as the cathode material and a graphitic carbon electrode with a metallic backing as the anode^53–55^. Although LFP batteries have a slightly lower energy density compared to other Li-ion cell chemistries due to their lower operating voltage, their special features, such as low cost, low toxicity, low self-discharge, high cycle life, high power, and high thermal stability, make them finds many roles in vehicle usage^56–58^, utility-scale stationary application^59–61^, and backup power^62–64^. The test procedures are designed according to UL 1974 and used to evaluate the safety and performance of the repurposed LFP batteries. The charge and discharge profile datasets provide researchers and engineers the characteristic curves to estimate the quality of repurposed batteries. Moreover, the profile datasets can be used in the model-based engineering of repurposed battery cells, e.g., fitting the variables of an empirical model or validating the results of a theoretical model.

## Methods

The UL 1974 standard^51,52^ covers the sorting and grading processes of battery packs, modules, and cells as well as electrochemical capacitors that were originally configured and used for other purposes, such as EV propulsion^65–67^, vehicle auxiliary power^68–70^, and light electric rail applications^71–73^. Furthermore, the focused purposes intend for a repurposed application, such as for use in energy storage systems^74–76^ and other applications for battery packs, modules, cells, and electrochemical capacitors. This standard also covers application-specific requirements for repurposed battery systems and battery systems utilizing repurposed modules, cells, and other components. (This standard does not include the process for remanufactured batteries, also referred to as refurbished or rebuilt batteries.)

The battery module can be decomposed into cells and used components according to UL 1974. The used components of the battery systems, such as the battery enclosure, battery management system (BMS), thermal management systems, and other auxiliary systems, should not be considered for repurposing if they have already been used longer than the calendar expiration date specified by the original manufacturer. The cells preparing for repurposing will undergo the performance test for sorting. UL 1974 suggests that the following test procedures shall be conducted by the repurposed manufacturer as part of the routine analysis of the incoming battery assembly:

1. Incoming open circuit voltage (OCV) measurements (Sec. 19.2 of UL 1974)
2. Incoming high voltage isolation check (Sec. 19.3 of UL 1974)
3. Capacity check (Sec. 19.4 of UL 1974)
4. Internal resistance check (Sec. 19.5 of UL 1974)
5. Check of BMS controls and protection components (Sec. 19.6 of UL 1974)
6. Discharge/charge cycle test (Sec. 19.7 of UL 1974)
7. Self-discharge (Sec. 19.8 of UL 1974)

### Charge and discharge profile measurement according to UL 1974

The charge and discharge profile measurement according to Sec. 19 of UL 1974 is divided into two primary procedures. The first procedure with detailed steps containing Secs. 19.2 and 19.4 of UL 1974 are listed in Table 1. The second procedure with detailed steps containing Secs. 19.5, 19.7, and 19.8 of UL 1974 are listed in Table 2. The key parameters in the procedures are described as follows.

**Table 1:** Test procedure 1 of charge and discharge profile measurement.

Procedure and Step	Action	Key Parameters	Description	As per UL 1974
P1S1	Rest	${t}_{rest}=1$ minute	Rest for 1 minute, observing the stability of OCV, and obtaining $OC{V}_{ini}$	19.2
P1S2	Charge in CC-CV mode	CC: ${I}_{const}=0.05Ca{p}_{N}/{\rm{h}}$ CV: ${V}_{thres}=3.5$ V, ${I}_{cut}=0.045Ca{p}_{N}/{\rm{h}}$	Safe charge at small ${C}_{R}=0.05{{\rm{h}}}^{-1}$	N/A
P1S3	Charge in CC-CV mode	CC: ${I}_{const}=0.1Ca{p}_{N}/{\rm{h}}$ CV: ${V}_{thres}=3.5$ V, ${I}_{cut}=0.095Ca{p}_{N}/{\rm{h}}$	Safe charge at small ${C}_{R}=0.1{{\rm{h}}}^{-1}$	N/A
P1S4	Charge in CC-CV mode	CC: ${I}_{const}=0.2Ca{p}_{N}/{\rm{h}}$ CV: ${V}_{thres}=3.5$ V, ${I}_{cut}=0.195Ca{p}_{N}/{\rm{h}}$	Safe charge at small ${C}_{R}=0.2{{\rm{h}}}^{-1}$	N/A
P1S5	Charge in CC-CV mode	CC: ${I}_{const}=0.5Ca{p}_{N}/{\rm{h}}$ CV: ${V}_{thres}=3.5$ V, ${I}_{cut}=0.05Ca{p}_{N}/{\rm{h}}$	Full charge at ${C}_{R}=0.5{{\rm{h}}}^{-1}$	19.4
P1S6	Rest	${t}_{rest}=1$ hour	Rest for 1 hour	19.4
P1S7	Discharge in CC mode	CC: ${I}_{const}=0.5Ca{p}_{N}/{\rm{h}}$, ${V}_{cut}=2.5$ V	Full discharge at ${C}_{R}=0.5{{\rm{h}}}^{-1}$ and obtaining $Ca{p}_{D}$	19.4
P1S8	Rest	${t}_{rest}=1$ hour	Rest for 1 hour	19.4
P1S9	Charge in CC-CV mode	CC: ${I}_{const}=0.5Ca{p}_{N}/{\rm{h}}$ CV: ${V}_{thres}=3.5$ V, ${I}_{cut}=0.05Ca{p}_{N}/{\rm{h}}$	Full charge at ${C}_{R}=0.5{{\rm{h}}}^{-1}$ and obtaining $Ca{p}_{C}$	19.4
P1S10	Rest	${t}_{rest}=1$ hour	Rest for 1 hour	19.4

The procedure is designed according to UL 1974. The notation “P *n* S *m*” indicates “the *m*-th step of the test procedure *n*.”

**Table 2:** Test procedure 2 of charge and discharge profile measurement.

Procedure and Step	Action	Key Parameters	Description	As per UL 1974
P2S1	Rest	${t}_{rest}=1$ minute	Rest for 1 minute, and observing the stability of OCV	N/A
P2S2	Charge in CC-CV mode	CC: ${I}_{const}=0.5Ca{p}_{RX}/{\rm{h}}$ CV: ${V}_{thres}=3.5$ V, ${I}_{cut}=0.05Ca{p}_{RX}/{\rm{h}}$	Full charge at ${C}_{R}=0.5{{\rm{h}}}^{-1}$	19.5
P2S3	Rest	${t}_{rest}=1$ hour	Rest for 1 hour	19.5
P2S4	Discharge in CC mode	CC: ${I}_{const}=0.2Ca{p}_{RX}/{\rm{h}}$, ${t}_{s}\ge {t}_{1}$	Two-tier DC load method at ${\rm{SOC}}=85 \%$ and obtaining ${V}_{85,1}$ and ${I}_{85,1}$	19.5
P2S5	Discharge in CC mode	CC: ${I}_{const}=1Ca{p}_{RX}/{\rm{h}}$, ${t}_{s}={t}_{2}$	Two-tier DC load method at ${\rm{SOC}}=85 \%$ and obtaining ${V}_{85,2}$ and ${I}_{85,2}$	19.5
P2S6	Discharge in CC mode	CC: ${I}_{const}=0.5Ca{p}_{RX}/{\rm{h}}$	Discharge to ${\rm{SOC}}=20 \%$	19.5
P2S7	Rest	${t}_{rest}=1$ hour	Rest for 1 hour	19.5
P2S8	Discharge in CC mode	CC: ${I}_{const}=0.2Ca{p}_{RX}/{\rm{h}}$, ${t}_{s}={t}_{1}$	Two-tier DC load method at ${\rm{SOC}}=20 \%$ and obtaining ${V}_{20,1}$ and ${I}_{20,1}$	19.5
P2S9	Discharge in CC mode	CC: ${I}_{const}=1Ca{p}_{RX}/{\rm{h}}$, ${t}_{s}={t}_{2}$	Two-tier DC load method at ${\rm{SOC}}=20 \%$ and obtaining ${V}_{20,2}$ and ${I}_{20,2}$	19.5
P2S10	Discharge in CC mode	CC: ${I}_{const}=0.5Ca{p}_{RX}/{\rm{h}}$, ${V}_{cut}=2.5$ V	Full discharge at ${C}_{R}=0.5{{\rm{h}}}^{-1}$	N/A
P2S11	Rest	${t}_{rest}=1$ hour	Rest for 1 hour	N/A
P2S12	Charge in CC-CV mode	CC: ${I}_{const}=0.5Ca{p}_{RX}/{\rm{h}}$ CV: ${V}_{thres}=3.5$ V, ${I}_{cut}=0.05Ca{p}_{RX}/{\rm{h}}$	Full charge at ${C}_{R}=0.5{{\rm{h}}}^{-1}$ and obtaining $Ca{p}_{C1}$	19.7
P2S13	Rest	${t}_{rest}=1$ hour	Rest for 1 hour	19.7
P2S14	Discharge in CC mode	${I}_{const}=0.5Ca{p}_{RX}/{\rm{h}}$, ${V}_{cut}=2.5$ V	Full discharge at ${C}_{R}=0.5{{\rm{h}}}^{-1}$ and obtaining $Ca{p}_{DN}$	19.7
P2S15	Rest	${t}_{rest}=1$ hour	Rest for 1 hour	19.7
P2S16	Charge in CC-CV mode	CC: ${I}_{const}=0.5Ca{p}_{RX}/{\rm{h}}$ CV: ${V}_{thres}=3.5$ V, ${I}_{cut}=0.05Ca{p}_{RX}/{\rm{h}}$	Full charge at ${C}_{R}=0.5{{\rm{h}}}^{-1}$ and obtaining $Ca{p}_{C2}$	19.7
P2S17	Rest	${t}_{rest}=1$ hour	Rest for 1 hour.	19.7
P2S18	Discharge in CC mode	${I}_{const}=1Ca{p}_{RX}/{\rm{h}}$, ${V}_{cut}=2.5$ V	Full discharge at ${C}_{R}=1{{\rm{h}}}^{-1}$ and obtaining $Ca{p}_{DM}$	19.7
P2S19	Rest	${t}_{rest}=1$ hour	Rest for 1 hour	N/A
P2S20	Charge in CC-CV mode	CC: ${I}_{const}=0.5Ca{p}_{RX}/{\rm{h}}$ CV: ${V}_{thres}=3.5$ V, ${I}_{cut}=0.05Ca{p}_{RX}/{\rm{h}}$	Full charge at ${C}_{R}=0.5{{\rm{h}}}^{-1}$ and obtaining $Ca{p}_{C3}$	19.8
P2S21	Rest	${t}_{rest}=5$ minutes	Rest for 5 minutes after P2S20 and obtaining $OC{V}_{5{\rm{m}}}$	19.8
P2S22	Rest	${t}_{rest}=55$ minutes	Rest for 1 hour after P2S20 and obtaining $OC{V}_{1{\rm{h}}}$	19.8
P2S23	Rest	${t}_{rest}=23$ hours	Rest for 24 hours after P2S20 and obtaining $OC{V}_{24{\rm{h}}}$	19.8

In the incoming open circuit voltage (OCV) measurements (P1S1 in Table 1), the OCVs of cells ($OC{V}_{ini}$) are measured. The measured OCVs shall be compared to the minimum voltage limit acceptable for the cell specified by the repurposed manufacturer, e.g., $2.5{\rm{V}}\le OC{V}_{ini}\le 3.5{\rm{V}}$ for LFP battery cell in this work. In addition, the OCVs are measured for a period of time (${t}_{rest}=1$ minute) to further check the stability of the OCV. The incoming high voltage isolation check is ignored, since the battery module is decomposed into cells. The insulation breakdown check of the battery system becomes unnecessary. Three charge steps with small current rates (P1S2–P1S4 in Table 1) are added into the procedure for slow and safe charging. The cell is charged in standard CC-CV mode with constant current ${I}_{const}={C}_{R}Ca{p}_{N}$, threshold voltage ${V}_{thres}=3.5$ V, and cutoff current ${I}_{cut}=({C}_{R}-0.005{{\rm{h}}}^{-1})Ca{p}_{N}$, where ${C}_{R}\equiv I/Ca{p}_{N}$, also called C-rate, is the current *I* per unit of nominal ampere hour capacity $Ca{p}_{N}$. The chosen C-rates in P1S2, P1S3, and P1S4 are 0.05 h^−1^, 0.1 h^−1^, and 0.2 h^−1^, respectively. The charge current is gradually increased to avoid abnormal voltage raising. (The details of standard charge and discharge processes are stated in the following subsection).

The capacity check of the battery cell according to the instructions of Sec. 19.4 of UL 1974 is designed as follows (P1S5–P1S10 in Table 1). The cell is fully charged by the standard CC-CV charge process under conditions ${I}_{const}=0.5Ca{p}_{N}/{\rm{h}}$ (i.e., ${C}_{R}=0.5$), ${V}_{thres}=3.5$ V, ${I}_{cut}=0.05Ca{p}_{N}/{\rm{h}}$. Then, the cell is fully discharged by the standard CC discharge process under conditions ${C}_{R}=0.5$ and discharge cutoff voltage ${V}_{cut}=2.5$ V. The discharge ampere hour capacity $Ca{p}_{D}$ is obtained after the full discharge process. At last, the cell is fully charged again for the next test, and the charge ampere hour capacity $Ca{p}_{C}$ is also obtained, where the charge (discharge) ampere hour capacity is calculated by integrating the current *I* over the full charge time *t*_*c*_ (the full discharge time *t*_*d*_), i.e., $Ca{p}_{C}(Ca{p}_{D})={\int }_{0}^{{t}_{c}({t}_{d})}|I(\tau )|d\tau$. The rest time between the charge and discharge processes is one hour.

The battery cells require capacity sorting before the next procedure. The obtained discharge ampere hour capacity of the repurposed battery cell is usually small than the nominal ampere hour capacity, i.e., $Ca{p}_{D}\le Ca{p}_{N}$. The battery cell shall be sorted into various groups ($Ca{p}_{RX}\le Ca{p}_{D} < Ca{p}_{R(X+\Delta X)}$) according to the value of *Cap*_*D*_, where $Ca{p}_{RX}=(X/100)Ca{p}_{N}$ is the remaining ampere hour capacity and $X\in {\mathbb{R}}$ is a positive real number. For example, when the battery cell is in the $Ca{p}_{R80}\le Ca{p}_{D} < Ca{p}_{R85}$ capacity group, its discharge capacity is greater than or equal to 80% of $Ca{p}_{N}$ and less-than 85% of $Ca{p}_{N}$. In this work, *X* = 100, 95, 90, 85, 80, …, 10, 5, 0, and $\Delta X=5$ is used to cover all capacity range without gap and overlap. It should be noted that the current rate (${C}_{R}$) in procedure 2 is based on the remaining ampere hour capacity ($Ca{p}_{RX}$) instead of the nominal ampere hour capacity ($Ca{p}_{N}$) in procedure 1.

The internal resistance check following the instruction of Sec. 19.5 of UL 1974 is listed in P2S2–P2S9 in Table 2. After the one-minute rest (P2S1), full charge at ${C}_{R}=0.5{{\rm{h}}}^{-1}$ (P2S2), and one-hour rest (P2S3), the internal resistance is measured under CC-mode discharge by two-tier direct current (DC) load method at two different states of charge. State of charge (SOC) as an indicator for the remaining capacity ratio of the battery is defined as ${\rm{SOC}}(t)={\rm{SOC}}({t}_{0})-Ca{p}^{-1}{\int }_{{t}_{0}}^{t}I(\tau )d\tau$, where ${\rm{SOC}}({t}_{0})$ is the previous SOC of the battery, $Cap$ is the ampere hour capacity of the fully charged battery, and $I(\tau )$ is the current with positive (negative) value for discharge (charge)^77,78^. The $Cap$ could be chosen as the nominal ampere hour capacity ($Ca{p}_{N}$), the latest capacity, or the capacity at a given time for a specific purpose. (The details of the two-tier DC load method are described in the following subsection.) The battery cell is discharged to SOC = 85% under the current rate ${C}_{R}=0.2{{\rm{h}}}^{-1}$ (P2S4), as well as the voltage ${V}_{85,1}$ and the current ${I}_{85,1}$ are recorded at the end of this step. Then, the discharge current rate is changed to ${C}_{R}=1{{\rm{h}}}^{-1}$ (P2S5), and ${V}_{85,2}$ and ${I}_{85,2}$ are measured at the end of this step, where the time duration of the first tier ${t}_{1}=10{t}_{2}$ should smaller than the step time *t*_*s*_ and the time duration of the second tier *t*_2_ = 100 seconds is equal to the step time. The internal resistance ${R}_{85}$ at ${\rm{SOC}}=85 \%$ can be calculated by Eq. 1. After the cell is discharged to ${\rm{SOC}}=20 \%$ under ${C}_{R}=0.5{{\rm{h}}}^{-1}$ (P2S6) and rest for one hour (P2S7), the cell is discharged under ${C}_{R}=0.2{{\rm{h}}}^{-1}$ for ${t}_{s}={t}_{1}$ (P2S8), obtaining the voltage ${V}_{20,1}$ and the current ${I}_{20,1}$ at the end of this step. Then, ${C}_{R}$ is changed to $1{{\rm{h}}}^{-1}$ for ${t}_{s}={t}_{2}$ (P2S9). ${V}_{20,2}$ and ${I}_{20,2}$ are measured at the end of this step, and the internal resistance ${R}_{20}$ at ${\rm{SOC}}=20 \%$ can be calculated.

The discharge and charge cycle tests under normal and maximum loadings according to Sec. 19.7 of UL 1974 begin after the full discharge at ${C}_{R}=0.5{{\rm{h}}}^{-1}$ (P2S10) and the rest for one hour (P2S11). In the first cycle of charge and discharge, the cell is fully charged at ${C}_{R}=0.5{{\rm{h}}}^{-1}$ (P2S12) to get the charge ampere hour capacity of the 1st cycle $Ca{p}_{C1}$, and then fully discharged at ${C}_{R}=0.5{{\rm{h}}}^{-1}$ (P2S14) to obtain the discharge ampere hour capacity under normal loading $Ca{p}_{DN}$. In the second cycle, the cell is fully charged at ${C}_{R}=0.5{{\rm{h}}}^{-1}$ (P2S16) to get the charge ampere hour capacity of the 2nd cycle $Ca{p}_{C2}$, and then fully discharged at ${C}_{R}=1{{\rm{h}}}^{-1}$ (P2S18) to obtain the discharge ampere hour capacity under maximum loading $Ca{p}_{DM}$. The rest time between charge and discharge processes is one hour.

The self-discharge test as part of the determination of the state of health (Sec. 19.8 of UL 1974) is shown in P2S20–P2S23 in Table 2. The OCV of the fully charged cell shall be recorded at 5 minutes ($OC{V}_{5m}$ in P2S21), 1 hour ($OC{V}_{1h}$ in P2S22), and 24 hours ($OC{V}_{24h}$ in P2S23) after charging ($Ca{p}_{C3}$ in P2S20).

In this work, the voltage ranging from 2.5 to 3.5 V is adopted for safe working of the repurposed LFP battery cells (i.e., *V*_*cut*_ = 2.5 V and *V*_*thres*_ = 3.5 V), which is narrower than the safe working voltage range of new LFP battery cells (2–3.65 V). The voltage range can be adjusted according to the manufacturer’s design. In addition, the designed test procedures based on UL 1974 can be used for other types of Li-ion repurposed batteries.

It should be noted that not all battery cells are appropriate for repurposing. Before module disassembly, the OCV check is suggested for an effective judgement. For the modules with OCVs in the normal working range, their cells possess the potential for repurposing. For the modules with OCVs outside the normal working range, their cells should be recycled directly, saving the cost and time of the measurement.

### Standard charge and discharge processes of Li-ion battery

**Fig. 1:**
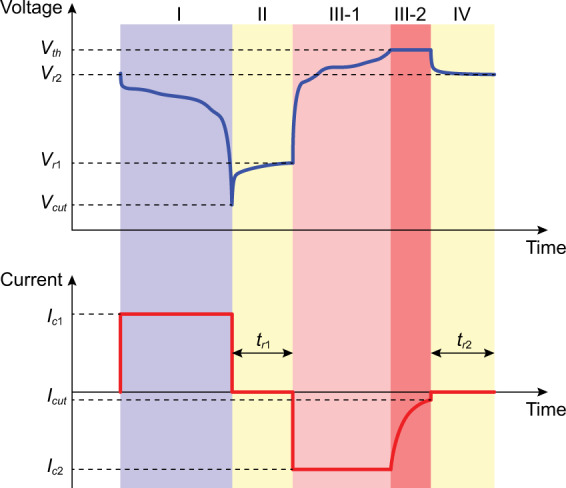
Standard charge and discharge processes of Li-ion battery. Step I (CC discharge): The battery is discharged at constant current ${I}_{c1}$ until the voltage drops to the cutoff voltage ${V}_{cut}$. Step II: Rest for the duration ${t}_{r1}$ without the current pass. The voltage gradually rises to ${V}_{r1}$. Step III-1 (CC charge): The battery is charged at constant current ${I}_{c2}$ until the voltage rises to the threshold voltage ${V}_{th}$. Step III-2 (CV charge): The battery is charged by maintaining ${V}_{th}$ until the current reaches the cutoff current ${I}_{cut}$. Step IV: Rest for the duration ${t}_{r2}$ without the current pass. The voltage gradually drops to ${V}_{r2}$.

There are four steps in the standard charge and discharge processes of Li-ion batteries. In the first step (as shown in the blue region I in Fig. 1), the battery is discharged under constant current ${I}_{c1}$, accompanied by a gradual voltage drop. As the voltage suddenly drops down to the cutoff voltage ${V}_{cut}$, the discharge process is terminated. The battery rests for the duration ${t}_{r1}$ in the second step, where no current passes through the battery and the voltage gradually rises to ${V}_{r1}$ (the yellow region II in Fig. 1). In the third step, the battery is charged under constant current ${I}_{c2}$ with a gradual voltage rise (the light red region III-1 in Fig. 1). When the voltage reaches the threshold value ${V}_{th}$, the battery keeps charging at the constant voltage ${V}_{th}$ by gradually lowering the charge current (the red region III-2 in Fig. 1). As the cutoff current ${I}_{cut}$ is reached, the charge process is completed. The battery rests for the duration ${t}_{r2}$ in the fourth step, where no current passes through the battery and the voltage gradually drops to ${V}_{r2}$ (the yellow region IV in Fig. 1). Based on the current and voltage constraints, the first and third steps are typically called the constant current (CC) discharge step and constant current-constant voltage (CC-CV) charge step, respectively.

The detailed charge and discharge processes might different for various manufacturers. Some differences are listed: (1) The order of charge and discharge steps could be exchanged. (2) The values of the discharge cutoff voltage $${V}_{cut}$$, the charge threshold voltage $${V}_{th}$$, and the charge cutoff current $${I}_{cut}$$. (3) The value of the discharge constant current $$| {I}_{c1}| $$ is not necessarily equal to the value of the charge constant current $$| {I}_{c2}| $$. (4) The signs for discharge and charge constant currents $$({I}_{c1},{I}_{c2})$$ might choose as (−, +), (+, −), or (+, +). (5) The rest duration $${t}_{r1}$$ is not necessarily equal to $${t}_{r2}$$. (6) The rest durations could set to zero, i.e., no step of rest.

### Two-tier DC load method

Direct current internal resistance (DCIR) of batteries indicates the resistance of current flowing through the battery. The value of DCIR is not fixed and varies depending on multiple factors, such as battery materials, type and concentration of electrolyte, temperature, as well as depth of discharge. The variation of DCIR has a great influence on battery discharge performance, especially for high power batteries. In general, the better the battery, the lower the internal resistance. Therefore, most battery manufacturers identify DCIR as a primary indicator for evaluating battery quality.

**Fig. 2:**
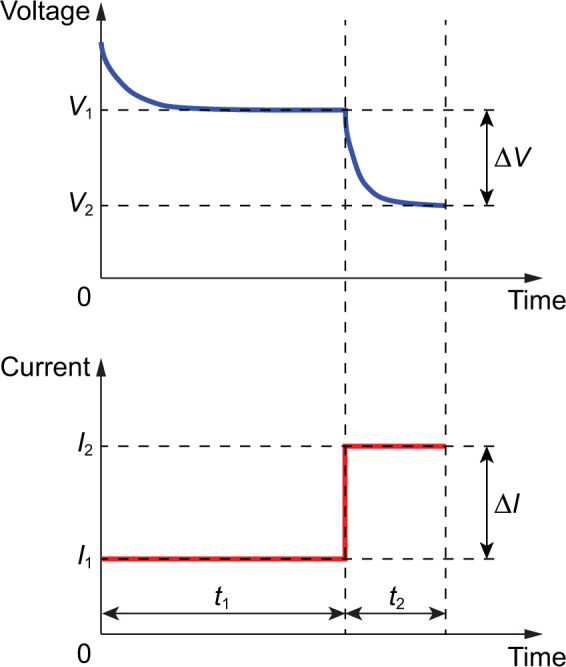
Two-tier DC load method for measuring the DCIR of batteries. The DC load test measures the battery’s internal resistance by reading the voltage drop. In the two-tier process, the DCIR is obtained by the Ohm’s law, dividing the voltage variation (${V}_{1}-{V}_{2}$) by the current variation (${I}_{2}-{I}_{1}$). The DC load test is the preferred method for evaluating the battery characteristic of DC power consumption.

Many techniques are applied to measure the DCIR of batteries, such as the tests conducted according to the IEC 61951-1 standard^79^, IEC 61960-3 standard^80^, and ISO 12405-4 standard^81^. In UL 1974, the two-tier DC load method is adopted, offering an alternative method by applying two sequential discharge loads of different currents and time durations. The battery first discharges at a lower constant current *I*_1_ for *t*_1_ seconds, dropping to a voltage *V*_1_, and then discharges at a higher constant current *I*_2_ for *t*_2_ seconds, dropping to a voltage *V*_2_ (as shown in Fig. 2). The DCIR, *R*_*DC*_, is obtained by the Ohm’s law as

1 $${R}_{DC}=\frac{\Delta V}{\Delta I}=\frac{{V}_{1}-{V}_{2}}{{I}_{2}-{I}_{1}}.$$

Some suggestions and comments from UL 1974: (1) The higher constant current is five times the lower one, i.e., *I*_2_ = 5*I*_1_. (2) Voltage and current during the discharge should be recorded at a rate not less than $10/{t}_{2}$ sample per second, i.e., $1/{t}_{rec}\ge 10/{t}_{2}$ (or ${t}_{rec}\le {t}_{2}/10$), where ${t}_{rec}$ is the data recording time interval. (3) Evaluating the voltage signature under the two load conditions offers additional information about the battery (the values are strictly resistive and do not reveal SOC or capacity estimations). (4) The load test is the preferred method for batteries that power DC loads.

### Measurement equipment and data collection

**Fig. 3:**
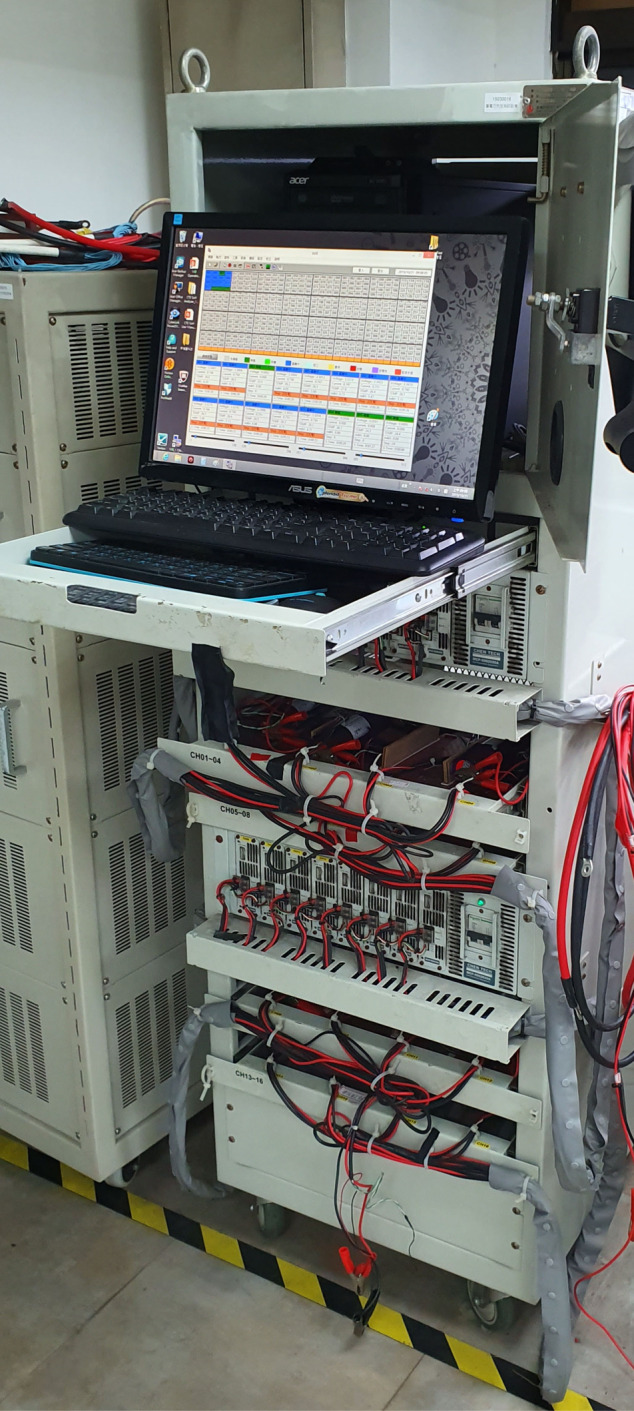
Battery test system. The CTE-MCP-5082020A battery test system (made by Chen Tech Electric Mfg. Co., Ltd., Taiwan) is used for evaluating the performance of the battery cells. There are 16 channels, and each of them can provide measurements of voltage, current, and temperature simultaneously.

The charge and discharge performance of the batteries were evaluated using the battery test system (CTE-MCP-5082020A, Chen Tech Electric Mfg. Co., Ltd., Taiwan) as shown in Fig. 3. The data was logged every ten seconds (${t}_{rec}=10$ sec) by the CTE-Will software (version 1.13tc). The environmental temperature was controlled at room temperature (25–32 °C). Output data was saved in the format of csv file, containing various information, including data point, step, step time (hh:mm:ss), voltage (V), current (A), power (W), temperature (°C), capacity (mAh), energy (Wh), total time (hh:mm:ss), and end status.

### First-life applications of the repurposed batteries

The first-life applications of these repurposed cells are power battery modules used in golf carts. The golf course is a relatively simple environment for design verification of the power battery. There are flat roads for continuous power output tests and some gentle slopes for the up and downhill tests. The power battery modules normally operate in two conditions: instant high power output (*C*_*R*_ = 3–6 h^−1^) for motor start and continuous medium power output (*C*_*R*_ = 1–3 h^−1^) for advancing the golf cart continuously. These battery modules have been used for 1–2 years, and then they reach the end-of-life (EOL).

## Data Records

The battery under repurposing is the 15 Ah 40138-LFP battery cell originally made by C-life Technologies, Inc., where 40138 indicates that the dimension of the cylinder battery cell (40 mm diameter and 138 mm height). The detailed specification of the battery cells is available in the data repository^82^. Also, a brief description of the module disassembly procedure is listed.

**Fig. 4:**
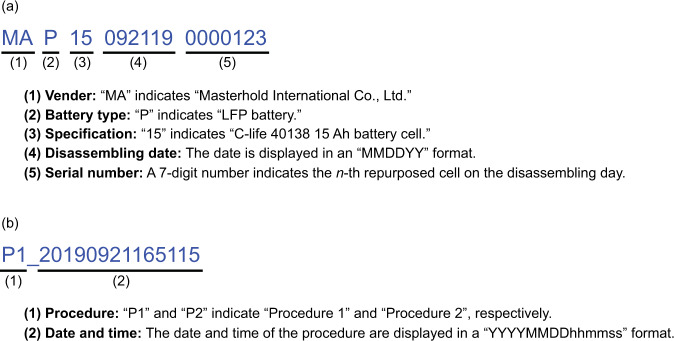
(**a**) Marking rule for the repurposed battery cell and the file folder in the database. (**b**) Naming rule for the charge and discharge profiles according to test procedures 1 and 2.

Each dataset exhibits the charge and discharge profiles of an individual run for a repurposed cell, which is available in the data repository^82^. An 18-digit code is used to mark the repurposed battery cell and the file folder of the dataset in the data repository, including the 2-digit vendor code, 1-digit battery type code, 2-digit specification code, 6-digit disassembling date code, and 7-digit serial number code (as shown in Fig. 4(a)). Each csv file provides the data of the charge and discharge profiles of the battery cell under the test procedures according to UL 1974. The csv file name is labeled by a 17-digit code, including the 2-digit procedure code, 14-digit date and time code (indicating the time of the start of the test), as well as 1-digit underline separating the two codes (as shown in Fig. 4(b)).

All datasets of repurposed cells from the modules possessing repurposed value (OCV within the normal working range) are included in the “Datasets of repurposed battery cells” folder of the data repository. There are 96 sets of data in total, without exclusion any of the cells in the modules for preserving the original distribution for further statistical or model analysis. On the other hand, some datasets of broken cells from the modules without repurposed value (OCV out of the normal working range) are listed in the “Datasets of broken battery cells” folder. These datasets are valuable for researchers to realize the behavior of the broken cells without taking the risk (e.g., thermal runaway) to do the test. Furthermore, the datasets provide some examples for researchers to recognize the abnormal behavior, and they can terminate the test while encountering similar behaviors, saving the experimental and engineering resources.

The metadata description of each column in the dataset is exhibited in Table 3. The data at the time stamp has two types: (1) current and (2) accumulated data. The former indicates that the data is measured at the time stamp, such as voltage, current, power, and temperature. The latter indicates that the data is the sum of the current and previous data, such as capacity and energy. Hence, the value of capacity (energy) stands for the amount of mAh (Wh) stored at the time stamp, and it will return to zero at the beginning of each step. The key values of all measurements obtained via procedures 1 and 2 are in the table available in the data repository^82^. The metadata description of each column in the dataset is shown in Table 4.

**Table 3:** Description of the metadata in the dataset.

Data	Unit	Description
Data point		*n*-th data point of the measurement
Step		Step number of the measurement
Step time	hh:mm:ss	Operating time of the step
Voltage	V	Measured voltage of the cell
Current	A	Measured current of the cell
Power	W	Calculated power of the cell
Temperature	°C	Measured temperature of the cell
Capacity	mAh	Calculated value for accumulated capacity stored in the cell
Energy	Wh	Calculated value for accumulated energy stored in the cell
Total time	hh:mm:ss	Total operating time of the measurement
End status		End status of the step. 0: The step is running. EC: The step is end by current constraint. EV: The step is end by voltage constraint. Time: The step is end by time constraint.

**Table 4:** Description of metadata for key values obtained via test procedures 1 and 2 according to UL 1974.

Data	Unit	Description	As per UL 1974
SN		Serial number of the cell	N/A
$OC{V}_{ini}$	V	Initial OCV	19.2 Re
$Ca{p}_{D}$	Ah	Discharge ampere hour capacity at ${C}_{R}=0.5{{\rm{h}}}^{-1}$	19.4 Re
$Ca{p}_{C}$	Ah	Discharge ampere hour capacity at ${C}_{R}=0.5{{\rm{h}}}^{-1}$	19.4 Ex
$X$ of $Ca{p}_{RX}$		Grouped by measured discharge ampere hour capacity $Ca{p}_{RX}$	19.4 Ex
${R}_{85}$	$\Omega$	DCIR obtained by two-tier DC load method at SOC = 85%	19.5 Re
${V}_{85,1}$	V	Measured voltage ${V}_{1}$ under the first tier at SOC = 85%	19.5 Re
${I}_{85,1}$	A	Measured current ${I}_{1}$ under the first tier at SOC = 85%	19.5 Re
${V}_{85,2}$	V	Measured voltage ${V}_{2}$ under the second tier at SOC = 85%	19.5 Re
${I}_{85,2}$	A	Measured current ${I}_{2}$ under the second tier at SOC = 85%	19.5 Re
${R}_{20}$	$\Omega$	DCIR obtained by two-tier DC load method at SOC = 20%	19.5 Re
${V}_{20,1}$	V	Measured voltage ${V}_{1}$ under the first tier at SOC = 20%	19.5 Re
${I}_{20,1}$	A	Measured current ${I}_{1}$ under the first tier at SOC = 20%	19.5 Re
${V}_{20,2}$	V	Measured voltage ${V}_{2}$ under the second tier at SOC = 20%	19.5 Re
${I}_{20,2}$	A	Measured current ${I}_{2}$ under the second tier at SOC = 20%	19.5 Re
$Ca{p}_{C1}$	Ah	Charge ampere hour capacity at ${C}_{R}=0.5{{\rm{h}}}^{-1}$	19.7 Ex
$Ca{p}_{DN}$	Ah	Discharge ampere hour capacity under normal loading conditions (${C}_{R}=0.5{{\rm{h}}}^{-1}$)	19.7 Re
$Ca{p}_{C2}$	Ah	Charge ampere hour capacity at ${C}_{R}=0.5{{\rm{h}}}^{-1}$	19.7 Ex
$Ca{p}_{DM}$	Ah	Discharge ampere hour capacity under maximum loading conditions (${C}_{R}=1{{\rm{h}}}^{-1}$)	19.7 Re
$Ca{p}_{C3}$	Ah	Charge ampere hour capacity at ${C}_{R}=0.5{{\rm{h}}}^{-1}$	19.7 Ex
$OC{V}_{5m}$	V	Measured OCV at 5 minutes after full charge	19.8 Re
$OC{V}_{1h}$	V	Measured OCV at 1 hour after full charge	19.8 Re
$OC{V}_{24h}$	V	Measured OCV at 24 hours after full charge	19.8 Re

The word “Re” (“Ex”) in the “As per UL 1974” column indicates that the key value is required by UL 1974 (extended data of UL 1974).

## Technical Validation

**Fig. 5:**
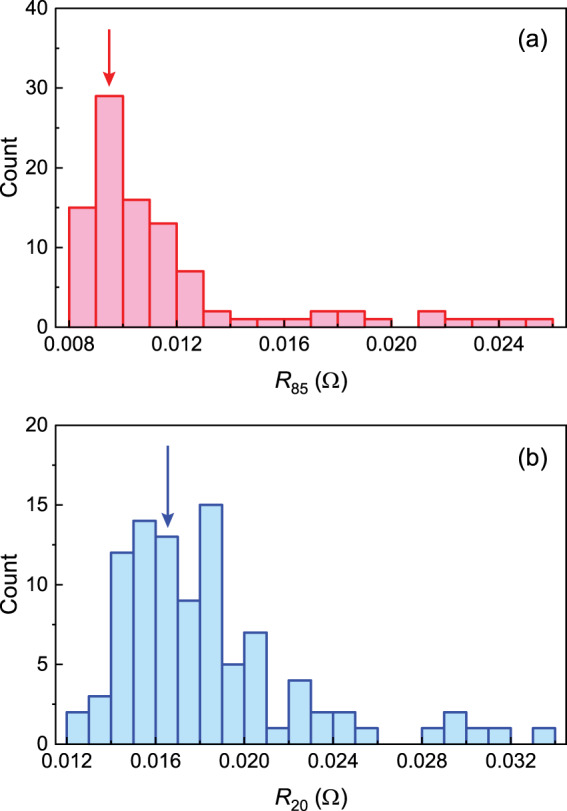
Central tendency of the DCIRs *R*_85_ and *R*_20_. The distributions of *R*_85_ and *R*_20_ exhibit the central tendency. (**a**) For ${R}_{85}$, most values locate around the central value 0.0095 Ω (red arrow). (**b**) For ${R}_{20}$, most values locate around the central value 0.0165 Ω (blue arrow).

In order to measure the voltage and current with high precision, the four-probe method is adopted (detailed description in the following subsection). The instrument is calibrated every year to guarantee the stability and precision of the measurements. For avoiding instability, an electric meter is used to randomly test the accuracy of voltage and current. From the statistical point of view, the key values obtained via test procedures 1 and 2 (in the table available in the repositories^82^) should exhibit the central tendency. For example, the DCIRs obtained by the two-tier DC load method at SOC = 85% and 20% locate around 0.0095 Ω and 0.0165 Ω, respectively (*R*_85_ and *R*_20_ in Fig. 5). The test procedures according to UL 1974 provide a reliable method for evaluating the repurposed battery cells.

### Four-probe method and temperature measurement

**Fig. 6:**
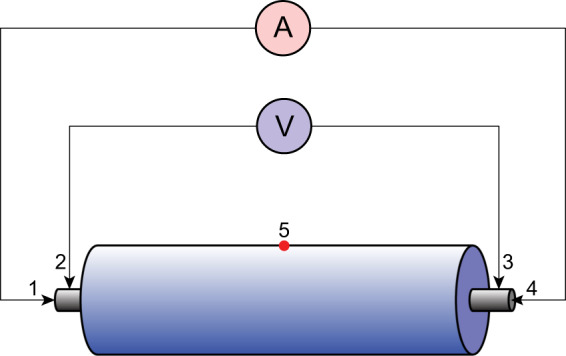
Four-probe method and temperature measurement. A current is passed through the outer probes (contacts 1 and 4) and induces a voltage in the inner voltage probes (contacts 2 and 3). The current and voltage can be measured simultaneously with high accuracy during the charge and discharge processes. On the other hand, the thermocouple locates at the center of the cylindrical surface of the cell for temperature measurement (red dot 5).

Four-probe method (also known as four-terminal sensing (4 T sensing), four-wire sensing, or four-point probes method) is an electrical resistance (impedance) measuring technique that uses separate pairs of current-carrying and voltage-sensing electrodes (as shown in Fig. 6). Separation of current and voltage electrodes eliminates the lead and contact resistance from the measurement, providing an advantage for precise measurement of low resistance values, making more accurate measurements than the simpler and more usual two-terminal (2 T) sensing. (When using 2 T sensing, the contact resistance at the point of measurement probe contact can reach several ohms or even dozens of ohms depending on environmental conditions.) In measuring the charge and discharge profiles of the battery, the four-probe method can provide high-accuracy voltage and current simultaneously for evaluating the battery quality.

The temperature is measured by the type K (chromel-alumel) thermocouples attaching above the geometric center of the cell. In this study, one thermocouple is attached at the center of the cylindrical surface of the cell (as shown in Fig. 6).

## Usage Notes

The test procedures 1 and 2 according to UL 1974 are designed for general-propose usage, i.e., the procedures could be used in testing LFP batteries and other types of secondary batteries. The profile datasets provided in this work can be used in the model-based engineering of repurposed battery cells: either to fit the variables of an empirical model or to validate the results of a theoretical model. The study involves no privacy or safety controls on public access to the data, i.e., everyone can access the data without limitations on data use.

## Data Availability

The datasets as reported are generated from experiments and are not relevant to any computer codes.
